# Inversion Reveals Perceptual Asymmetries in the Configural Processing of Human Body

**DOI:** 10.3389/fnbeh.2017.00126

**Published:** 2017-07-05

**Authors:** Daniele Marzoli, Chiara Lucafò, Caterina Padulo, Giulia Prete, Laura Giacinto, Luca Tommasi

**Affiliations:** Department of Psychological Sciences, Health and Territory, University of ChietiChieti, Italy

**Keywords:** human body, handedness, perceptual asymmetries, configural processing, inversion effect

## Abstract

Ambiguous human bodies performing unimanual/unipedal actions are perceived more frequently as right-handed/footed rather than left-handed/footed, which suggests a perceptual and attentional bias toward the right side of others’ body. A bias toward the right arm of human bodies could be adaptive in social life, most social interactions occurring with right-handed individuals, and the implicit knowledge that the dominant hand of humans is usually placed on their right side might also be included in body configural information. Given that inversion disrupts configural processing for human bodies, we investigated whether inversion reduces the bias toward the right side of human bodies. Consistent with our hypothesis, when presented with ambiguous stimuli depicting humans performing lateralized actions or movements, participants perceived a greater proportion of right-handed figures when the stimuli were shown upright than when the stimuli were shown inverted. The present findings seem to confirm our hypothesis that body configural information may include some form of knowledge about the probable handedness of other individuals, although alternative accounts involving the role of experience cannot be ruled out.

## Introduction

In a recent study, we found that when required to indicate the orientation (front or back view) of pictures of ambiguous human silhouettes performing one-handed manual actions, both right- and left-handers perceived the figures more frequently in an orientation congruent with a movement performed with the right rather than the left hand (Marzoli et al., 2015; see Lucafò et al., 2016 for consistent results for foot actions). This result suggests the presence in both right- and left-handers of a perceptual and attentional bias toward the right side of others’ body, and is in line with several studies dealing with the perception of sport actions, indicating that the outcomes of movements performed by right-handed or right-footed individuals are anticipated better than those of movements performed by left-handed or left-footed individuals (McMorris and Colenso, 1996; Hagemann, 2009; Loffing et al., 2012; Schorer et al., 2012). A bias toward the right arm of human bodies, likely due to a perceptual frequency effect (Faurie and Raymond, 2005), could be adaptive in social life, most social interactions occurring with right-handed individuals (see Marzoli et al., 2014 for a more detailed discussion). In particular, such a bias might imply an increased efficiency in monitoring both communicative and aggressive acts, the right limb being more used than the left in both types of behavior.

We have already proposed (Marzoli et al., 2014) that body configural information might include the implicit knowledge (in terms of both first-order relational information and structural information; Reed et al., 2006) that the dominant hand of humans is usually placed on their right side. Inversion disrupts configural processing for human bodies (e.g., Reed et al., 2003, 2006), and the effect is reduced in individuals with autism (Reed et al., 2007), who are known to exhibit impaired configural processing (Behrmann et al., 2006). Therefore, in the present study we aimed to investigate whether inversion reduces the bias toward the right side of human bodies. To this aim, we tested whether when presented with ambiguous stimuli depicting humans performing lateralized actions or movements, participants would have perceived a greater proportion of right-handed figures when the stimuli were shown upright than when the stimuli were shown inverted. Moreover, in order to assess whether the effect is generalizable across different types of stimuli, we tested participants with three different tasks. In the first task, we used the same stimuli as those used in Marzoli et al. (2015). In the second task, we used stimuli that required the same response (perceived front or back view) as those in the first task, but that were physically different (dynamic point-light figures). In the third task, we used stimuli that required a different response (perceived clockwise or counterclockwise rotation) and that were physically different (rotating ambiguous silhouettes), but for which we could nonetheless expect a bias toward the right limb on the basis of our previous study with the spinning dancer illusion (Lucafò et al., 2016). We believe that any conclusion about the effects of inversion on the bias toward the right side of human bodies would be strengthened if results can be generalized across different types of stimuli. Given that our previous studies (Marzoli et al., 2015; Lucafò et al., 2016) indicate that such a bias is comparable across different types of stimuli and response modalities, we predicted that inversion effects would have been similar in the different tasks.

## Materials and Methods

Following the recommendations of Simmons et al. (2012), we report how we determined our sample size, all data exclusions, all manipulations and all measures in the study.

### Participants

Given that: (1) the present study involved an additional manipulation (upright vs. inverted condition) with respect to our previous study with ambiguous human silhouettes performing one-handed manual actions (Marzoli et al., 2015); (2) we could not predict whether inversion would abolish or only reduce the bias toward the right side of human bodies; and (3) the high likelihood that some participants would not have completed the whole experiment (consisting of two separate sessions), we decided to schedule the recruitment of twice as many participants as those tested in that study (see Supplementary Table S1 for more detailed information). Thus, 47 participants (23 females and 24 males; age: 19–39 years) were initially recruited. Five participants (1 female and 4 males) who completed only the first session and three participants (1 female and 2 males) who reported awareness or suspicion about the experimental hypotheses or manipulations were excluded. We further excluded eight participants (4 females and 4 males) who gave the same response (“FRONT” or “BACK” in Tasks 1 and 2; “CLOCKWISE” or “COUNTERCLOCKWISE” in Task 3) in any session of any task, because this might indicate less than full engagement with the task. According to the laterality score obtained in the Italian version of the Edinburgh Handedness Inventory (Salmaso and Longoni, 1985), the remaining 31 participants (17 females and 14 males; age: 19–38 years) were classified as right-handers (28 subjects with a positive laterality score [range: 0.13/1.00; *M* = 0.67 ± 0.041 SEM]) or left-handers (3 subjects with a negative laterality score [range: −0.71/−0.40; *M* = −0.56 ± 0.091 SEM]). All the participants had normal or corrected-to-normal vision.

### Stimuli

#### Upright Session

##### Task 1

**Figure 1:**
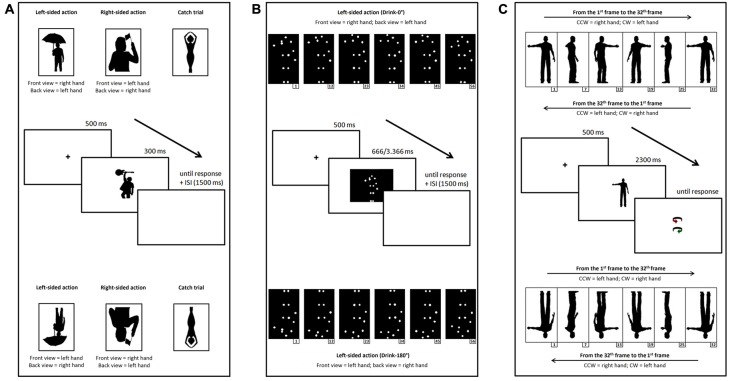
Examples of upright (top) and inverted (bottom) stimuli and schematic representation of the time course of a trial (center) for Task 1 **(A)**, 2 **(B)** and 3 **(C)**.

We used the same stimuli as in Marzoli et al. (2015), consisting of 26 silhouettes of female and male persons performing one-handed manual actions (such as smoking, drinking from a glass/bottle, holding something, waving a flag and so on) printed in black against a white background, and their mirror images. The original silhouettes (obtained by editing photographs and line drawings taken from the Web) were selected with the constraints that: (1) the action was clearly represented on the figure’s right or left side (from an observer’s perspective); and (2) the figure’s orientation (front or back view) was ambiguous (see Figure 1A for an example). Each original silhouette was mirrored horizontally in order to obtain a right-sided (from the observer’s perspective) action (congruent with a right- or left-handed action if the figure was perceived as back- or front-facing, respectively) and a left-sided action (congruent with a left- or right-handed action if the figure was perceived as back- or front-facing, respectively). Moreover, 26 silhouettes of female and male persons who were not performing one-handed manual actions (e.g., holding objects with both hands or not performing actions; see Figure 1A for an example) and the respective mirror images were used as catch trials. At a viewing distance of 57 cm, stimuli measured, on average, 6.5° horizontally and 10.4° vertically.

##### Task 2

As stimuli, we used seven movies (Drink-0°, Mow-0°, Paint-0°, Play Tennis-0°, Salute-0°, Saw-0°, Stir-0°) selected by the database of human point-light actions created by Vanrie and Verfaillie (2004; available at http://ppw.kuleuven.be/home/english/research/lep/images/resources/database), and their mirror images. Each stimulus of the database consists of a point-light action defined by 13 white markers (head, two shoulders, two elbows, two wrists, two hips, two knees and two ankles) against a black background. We selected stimuli in which the human figure was depicted with the torso approximately parallel to the projection plane and the dominant hand was clearly represented on the figure’s right or left side (from an observer’s perspective). Although Vanrie and Verfaillie define the selected orientation as “frontal view”, the figure’s orientation (front or back view) is actually ambiguous (see Figure 1B for an example). Each original movie was mirrored horizontally in order to obtain a right-sided (from the observer’s perspective) action (congruent with a right- or left-handed action if the figure was perceived as back- or front-facing, respectively) and a left-sided action (congruent with a left- or right-handed action if the figure was perceived as back- or front-facing, respectively). Moreover, seven movies (Crawl-0°, Cycle-0°, Drive-0°, Jump-0°, Row-0°, Walk-0°, Wave-0°) displaying figures seen in “frontal view”, but not performing lateralized manual actions, and the respective mirror images were used as catch trials. At a viewing distance of 57 cm, stimuli measured, on average, 4.1° horizontally and 8.9° vertically.

##### Task 3

As stimuli, we used 128 animations obtained by an original animation, created with the software Poser Pro 2012 (Smith Micro Inc.), representing the silhouette of a human male who rotates about his vertical axis while maintaining a static posture (standing on both legs and with one arm close to the body and the other arm extended; Figure 1C). Given that the silhouette is depicted in black against a white background and given that no clear-cut depth cue is available, the animation is ambiguous and can be perceived as rotating either clockwise (a percept consistent with an extended left arm in the original animation) or counterclockwise (a percept consistent with an extended right arm in the original animation). Then, after decomposing the original animation in its 32 constituent frames, we created 64 different versions of the animation, each consisting of a complete rotation of the silhouette, by rearranging the 32 frames according to starting frame and order (i.e., from the 1st frame to the 32th and vice versa, from the 2nd frame to the 1st and vice versa, and so on). The order manipulation allowed to counterbalance the association between spinning direction and extended arm, because in the original order (which can be defined “palmward” when considering the hand movement) the clockwise rotation is congruent with an extended left arm, whereas in the reversed order (which can be defined “backward”) the clockwise rotation is congruent with an extended right arm, and vice versa for the counterclockwise rotation (Figure 1C). Although we set the Poser parameters (such as camera distance and elevation) so as to remove—as far as possible—potential perspective cues (such as relative size and relative height), we created a second set of 64 animations by mirroring horizontally each frame, a manipulation that allowed to counterbalance the effects of any remaining uncontrolled depth cue or asymmetry possibly biasing the perception of the extended arm. In sum, the final set of stimuli consisted of 128 animations representing each possible combination of starting frame, type of rotation (palmward movement or backward movement) and mirroring. At a viewing distance of 57 cm, the component frames of each stimulus measured around 10.8° vertically and, on average, around 5.1° horizontally.

#### Inverted Session

For each task, inverted versions of the stimuli used in the upright session were created by rotating them by 180° (Figure 1). In Tasks 1 and 2, the difference of interest was that a right-sided (from the observer’s perspective) action was congruent with a left- or right-handed action if the figure was perceived as back- or front-facing, respectively, whereas a left-sided action was congruent with a right- or left-handed action if the figure was perceived as back- or front-facing, respectively. In Task 3, the difference of interest was that the association between spinning direction and extended arm was reversed (in the palmward movement the clockwise rotation is congruent with an extended right arm, whereas in the backward movement the clockwise rotation is congruent with an extended left arm, and vice versa for the counterclockwise rotation).

### Procedure

Participants were tested in two sessions (one with upright stimuli and the other with inverted stimuli) on two consecutive days. For each task, the procedure was the same for both the upright and the inverted session. For each participant, the task order was the same in both sessions. The tasks were run using SuperLab 4.0 on a Windows notebook with an Intel processor and a 15.4-inch monitor. Participants were seated comfortably in a quiet room, with their eyes about 57 cm from the computer screen, and were required to place their hands palm-down on the table and not to cross their legs, arms or even fingers throughout the experiment.

Session order was counterbalanced across participants, and the combinations of task order, Task 1 block order, and Task 3 response arrow spinning direction (CW or CCW), color (red or green) and position (above or below) were pseudo-randomized across participants (so that each task order, each Task 1 block order, and each combination of Task 3 response arrow spinning direction, color and position were presented approximately to the same number of female and male subjects; see Supplementary Table S1 for more detailed information). At the end of the second session, in order to assess the participant’s hand preference, she/he was administered the Italian version of the Edinburgh Handedness Inventory (Salmaso and Longoni, 1985). Each participant was also probed for awareness of the purpose of the study, and when explicitly required, the experimenter debriefed the participant about the purpose. Since neither invasive nor risky procedures were involved and since the data were analyzed anonymously, participants were required to give only oral consent. The study was carried out in accordance with the principles of the Declaration of Helsinki and following the approval of the Ethics Committee for Biomedical Research of the University of Chieti and Pescara.

#### Task 1

Participants were administered 104 trials (52 target trials and 52 catch trials) in which a black fixation cross presented for 500 ms in the center of a white screen was followed by a black silhouette presented centrally for 300 ms and then by a completely white screen (Figure 1A). Participants were instructed to indicate the perceived orientation of the stimuli as fast as possible by pronouncing the words “FRONTE” (the Italian word for “FRONT”) or “SPALLE” (“BACK”). The experimenter, seated behind the participant, recorded the participant’s response by pressing the key “F” or “S” on a keyboard connected to the computer, and the following trial started after an interstimulus interval of 1500 ms. The 104 trials were arranged in two separate blocks (A and B), so that the right- and left-sided versions of each silhouette were shown in different blocks, thus precluding them from being presented one after the other. This expedient, along with the inclusion of catch trials, was aimed to prevent participants from focusing overtly on the relevant aspect of the task (handedness). Stimuli were presented in a random sequence within each block, and the order of block presentation was counterbalanced across participants. After completing the first block, participants were allowed to rest as long as they needed before starting the second block.

#### Task 2

Participants were administered 56 trials (28 target trials and 28 catch trials; each stimulus was presented twice) in which a black fixation cross presented for 500 ms in the center of a white screen was followed by a point-light action (whose duration ranged from 666 ms to 3366 ms for the target trials and from 1000 ms to 4000 ms for the catch trials) presented centrally and then by a completely white screen (Figure 1B). Participants were instructed to indicate the perceived orientation of the stimuli as fast as possible by pronouncing the words “FRONTE” (“FRONT”) or “SPALLE” (“BACK”). The experimenter, seated behind the participant, recorded the participant’s response by pressing the key “F” or “S” on a keyboard connected to the computer, and the following trial started after an interstimulus interval of 1500 ms. The 56 trials were arranged in two separate identical blocks, so that each version (right- and left-sided) of each stimulus was shown only once in each block, thus precluding them from being presented twice in the same block. As in Task 1, the inclusion of catch trials was aimed to prevent participants from focusing overtly on the relevant aspect of the task (handedness). Stimuli were presented in a random sequence within each block, and the order of block presentation was counterbalanced across participants. After completing the first block, participants were allowed to rest as long as they needed before starting the second block.

#### Task 3

Participants were administered 128 trials in which a black fixation cross presented for 500 ms in the center of a white screen was followed by one of the previously described stimuli presented centrally and then by a pair of colored arrows (representing the two possible spinning direction of the silhouette), one slightly above and one slightly below the center of the screen (Figure 1C). Participants were instructed to gaze at the fixation point and to indicate the perceived spinning direction of the silhouette by pronouncing the words “ROSSO” (“RED”) or “VERDE” (“GREEN”) depending on which arrow represented their percept. The experimenter, seated behind the participant, recorded the participant’s response by pressing the key “R” or “V” on a keyboard connected to the computer, and then the next trial started. Stimuli were presented in a random sequence. The first frame of each stimulus lasted 750 ms, whereas each of the remaining 31 frames lasted 50 ms. This expedient was aimed at reducing the possible carry-over of responses from trial to trial (e.g., see Liu et al., 2012, who found percept carry-over even after a 30 s break between the presentation of an ambiguous spinning body and the next one). As in a previous study with the spinning dancer illusion (Lucafò et al., 2016), we decided to collect participants’ responses by means of two colored arrows, each representing a possible spinning direction, rather than by means of simple vocal responses such as “ORARIO” (the Italian word for “CLOCKWISE”) and “ANTIORARIO” (“COUNTERCLOCKWISE”), because the latter response modality seems to be rather troublesome for participants (maybe due to their difficulty in labeling as clockwise or counterclockwise a rotation about an axis approximately parallel to their own body axis). Before the task, in order to familiarize participants with the response modality, they were administered a pretest in which they had to use the two response arrows to indicate the spinning direction of a black human silhouette containing perspective cues (e.g., the relative size of the hands in different positions). This pretest went on until the participant was able to match without fail each spinning direction with the corresponding arrow, and led to the exclusion from the study of the few subjects who failed to perform the task.

#### Methodological Considerations

We are well-aware that the method of data collection used in the present study, where the experimenter recorded the participant’s vocal response by pressing a key, could engender some doubts about the underlying reason. However, we believe that it is the best way to collect this kind of data because we have found that asking participants to respond by means of key presses would result in even more serious problems, the use of two fingers or hands inducing Simon-like effects involving effector side and silhouette sidedness (Marzoli et al., in preparation). It should be also pointed out that the experimenter was placed behind the participant’s body and had the mere assignment to look at the keyboard and to press the key corresponding to the participant’s response without looking at the screen. Moreover, in order not to mislead the experimenter, who was blind to the stimuli, participants were explicitly required to provide only a single response to each stimulus and not to amend it even if they believed that their first impression was wrong.

### Data Analysis

We aimed to examine whether participants perceived a larger proportion of right-handed figures in the upright session than in the inverted session. Moreover, in order to assess whether the effect of session was consistent across different tasks, the effect of task was also examined. Thus, we performed a repeated measures analysis of variance (ANOVA) on the percentage of figures perceived as right-handed, using participant’s sex (female or male) as between-subjects factor, and the task (static silhouettes, point-light actions or rotating man) and the session (upright or inverted) as within-subjects factors. When needed, *post hoc* *t*-tests were carried out in order to specify the significant differences. Because of the low number of left-handers, it was not possible to include handedness as an independent variable in the ANOVA performed, so laterality score was correlated with the proportion of figures perceived as right-handed in each session.

## Results

**Figure 2:**
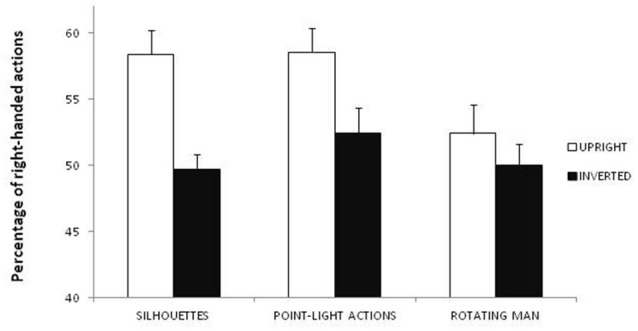
Percentage of figures perceived as right-handed according to stimuli (static silhouettes, point-light actions or rotating man) and condition (upright or inverted).

The ANOVA showed significant main effects of session (*F*_(1,29)_ = 11.107; *p* = 0.002; *η*^2^ = 0.101) and task (*F*_(2,58)_ = 4.861; *p* = 0.011; *η*^2^ = 0.034). As regards the effect of session, a larger proportion of figures were perceived as right-handed in the upright session (*M* = 56.4%) than in the inverted session (*M* = 50.7%; Figure 2). Moreover, one-sample two-tailed *t*-tests showed that in the upright session the figures were perceived more frequently as right-handed (*t*_(30)_ = 4.674; *p* < 0.001) than expected by chance (50%), whereas no difference was observed in the inverted session (*t*_(30)_ = 0.663; *p* = 0.512). As regards the effect of task, paired-sample two-tailed *t*-tests showed that a smaller proportion of figures were perceived as right-handed in Task 3 (rotating man: *M* = 51.2%) than in Task 1 (static silhouettes: *M* = 54%; *t*_(30)_ = −2.063; *p* = 0.048) and Task 2 (point-light actions: *M* = 55.5%; *t*_(30)_ = −2.894; *p* = 0.007; Figure 2). Moreover, one-sample two-tailed *t*-tests showed that the figures were perceived more frequently as right-handed than expected by chance (50%) in Task 1 (*t*_(30)_ = 3.812; *p* < 0.001) and Task 2 (*t*_(30)_ = 3.876; *p* < 0.001), but not in Task 3 (*t*_(30)_ = 1.472; *p* = 0.151). No significant correlation was observed between participants’ laterality score and the percentage of figures perceived as right-handed in either session.

## Discussion

The present findings seem to confirm our hypothesis that body configural information may include the knowledge that the dominant hand of humans is usually placed on their right side. Indeed, inversion, which is known to disrupt the configural processing of human bodies (e.g., Reed et al., 2003, 2006), abolished the bias to perceive right-handed actions found in the upright session. As predicted, and in line with our previous findings showing that the bias toward the right limb of human bodies is consistent across different types of stimuli and response modalities (Marzoli et al., 2015; Lucafò et al., 2016), the effect of session did not differ across the different tasks, which supports the robustness of our results. Our study thus provides an additional instance of the detrimental effects of body inversion on configural processing, such as reduction in perceptual grouping (Poljac et al., 2011, 2012), body posture recognition (Reed et al., 2003, 2006), action recognition (Dittrich, 1993) and emotion recognition from whole-body gestures and dance movements (Dittrich et al., 1996; Atkinson et al., 2007), as well as the abolishment of expertise-related advantage in action discrimination (Calvo-Merino et al., 2010). Moreover, a greater proportion of figures were perceived as right-handed in the tasks with static silhouettes and point-light actions than in the task with the rotating man. Although we found no interaction between session and task, it is plausible that upright rotating stimuli could elicit a weaker bias toward the right side of human bodies compared with upright stimuli with a fixed orientation (at least when the torso is approximately parallel to the projection plane as in the case of the static silhouettes and point-light actions used in the present study). In this regard, we would like to point out that in our previous work we observed a slightly larger bias toward the right side of human bodies when using static (53.6%; Marzoli et al., 2015) rather than rotating (51.7%; Lucafò et al., 2016) stimuli, although the role of methodological differences among the various studies (such as type and number of stimuli, sample size, and so on) cannot be discarded. Finally, it should be remarked that the significant effect of task should be considered with caution because of its small effect size.

Differently from our previous research on others’ action imagination, where a smaller bias toward the right side of bodies was observed in left-handers and in weak right-handers (Marzoli et al., 2011a,b, 2013, 2017), the fact that the preference for perceiving right-handed actions in the present study was not related to the degree of handedness in any session of any task suggests that implicit knowledge about human handedness might involve considerably more visual than motor processes (see Marzoli et al., 2014, 2015; Lucafò et al., 2016 for consistent findings and a more detailed discussion). In this regard, it is noteworthy that the asymmetry in configural visual processing of human bodies seems to parallel that reported for letters and digits. Indeed, whereas the naming and letter-digit discrimination of alphanumeric characters is rather unaffected by stimulus orientation (Corballis et al., 1978; White, 1980; Milivojevic et al., 2011), their mirror-normal discrimination (Cooper and Shepard, 1973; White, 1980; Milivojevic et al., 2011) and the discrimination of letters whose canonical forms are left-right mirror images of one another (i.e., “b” vs. “d” and “p” vs. “q”; Corballis and McLaren, 1984) is impaired when stimuli are rotated rather than upright. According to Cooper and Shepard (1973), the discrimination of mirror images—differently from naming and letter-digit discrimination—requires a holistic strategy involving mental rotation because it cannot be achieved by means of feature analysis. Similarly, in order to account for why disorientation disrupts the recognition of words more than that of letters and digits, Koriat and Norman (1989) suggested that orientation effects may be particularly strong when identification depends on the spatial arrangement of several elements. These interpretations are in agreement with the proposal by Jolicoeur (1990) that the identification of disoriented objects relies on at least two separate systems: a feature-based system (grounded on viewpoint-independent feature extraction) and a mental-rotation system (grounded on viewpoint-dependent configurations). Electroencephalographic recording during letter-digit and mirror-normal discrimination also indicates that feature extraction and mental rotation are temporally distinct processes, with the former and the latter occurring at the earliest and latest stages, respectively (Milivojevic et al., 2011). In our opinion, similarly to letters and digits, human-body configural information might include an asymmetrical representation of the dominant hand, with a well-defined left-right orientation. On the other hand, we want to point out that telling the right- or left-handedness of our target stimuli requires some sort of mental rotation that can be assimilated to the utmost stage of mirror-normal discrimination for alphanumeric characters, that is a mental rotation out of the picture plane (Milivojevic et al., 2011). We also believe that such an asymmetrical representation is weaker than that pertaining to alphanumeric characters both because around 10% of human beings consist of left-handers (e.g., see Coren, 1993) and because right-handers themselves occasionally use their left-hand. This could contribute to account for why the bias to perceive right-handed actions in upright stimuli (around 56% in the present study) is so distant from the actual predominance of right-handedness.

As we have already stressed in a recent review (Marzoli et al., 2014), although asymmetries in body configural representation could be linked to right-handedness at the level of phylogeny—because of the evolutionarily adaptive advantage of directing attention toward the region of visual space where others’ dominant hand usually operates—one could also wonder whether, at the ontogenetic level, frequent exposure to right-handed individuals may foster congruent biases. Therefore, it would be interesting to investigate whether an age-dependent increase in the asymmetrical representation of human bodies exists. In this regard, it could be hypothesized that people could not develop any asymmetrical representation for stimuli they have not been exposed to, so that the absence of any lateral bias for inverted ambiguous bodies in the present study might be accounted for by participants’ lack of experience with inverted human bodies, without calling upon the disruption of configural processing. Such an interpretation might also explain the fact that action and body posture recognition is significantly impaired under inversion (Dittrich, 1993; Dittrich et al., 1996; Reed et al., 2003, 2006; Atkinson et al., 2007; Calvo-Merino et al., 2010). Perhaps, discriminating between these alternative hypotheses would be possible by examining whether individuals with autism—who are known to be impaired in configural processing (Behrmann et al., 2006; Reed et al., 2007) but are unlikely to have reduced experience with right-handed individuals—exhibit a weaker effect of inversion on perceptual asymmetries for human bodies. Nonetheless, it should be remarked that the configural and experience accounts of the asymmetrical representation of human bodies are not necessarily mutually exclusive, recent research showing that learning and experience foster the emergence of configural processes such as perceptual grouping (Kimchi and Hadad, 2002; Yeh et al., 2003; Vickery and Jiang, 2009).

## Author Contributions

DM, CL, CP, GP and LT conceived the study. CL, CP and LG acquired data. DM and CL analyzed and interpreted data. DM and CL wrote the manuscript, and CP, GP, LG and LT revised it critically. All authors approve the submitted manuscript and agree to be accountable for the content of the work.

## Conflict of Interest Statement

The authors declare that the research was conducted in the absence of any commercial or financial relationships that could be construed as a potential conflict of interest.
