# Supplementary material for: Inversion Reveals Perceptual Asymmetries in the Configural Processing of Human Body
Source: Front Behav Neurosci. 2017 Jul 5;11:126. doi: 10.3389/fnbeh.2017.00126 (PMC5496943; doi:10.3389/fnbeh.2017.00126)
Supplement: Supplementary file 1 [file Table_1.doc]

Supplementary Material

# Inversion Reveals Perceptual Asymmetries in the Configural Processing of Human Body

Daniele Marzoli*, Chiara Lucafò, Caterina Padulo, Giulia Prete, Laura Giacinto, & Luca Tommasi

*** Correspondence:** Daniele Marzoli: [d.marzoli@unich.it](mailto:d.marzoli@unich.it)

**Participant Schedule**

We scheduled the recruitment of approximately twice as many participants as in our previous study with ambiguous human silhouettes performing one-handed manual actions (Marzoli et al., 2015), in which 24 participants were tested. For this purpose, we pseudo-randomly selected 12 out of the 48 possible combinations of task order, Task 1 block order, and Task 3 response arrow spinning direction (CW or CCW), color (red or green) and position (above or below), and assigned each to 4 participants (1 female and 1 male starting with the upright condition and 1 male and 1 female starting with the inverted condition). The extraction of the 12 experimental sequences was constrained so as to present each session order, each task order, each Task 1 block order, and each combination of Task 3 response arrow spinning direction, color and position to approximately the same number of female and male subjects. Because several participants deserted either the second or both sessions, an additional experimental sequence was selected. Finally, the second session was completed by 42 out of the 47 participants who completed the first session (see Supplementary Table 1 for a detailed list of the experimental sequences administered to the participants who completed both sessions).

# Supplementary Table

**Supplementary Table 1.** Detailed list of the experimental sequences administered to the participants who completed both sessions. Note: SS, Static Silhouettes/Task 1 (the numbers indicate the two different block orders); PLA, Point-Light Actions/Task 2; RM, Rotating Man/Task 3 (the numbers indicate the four different combinations of response arrow spinning direction, color and position); F_UPR, females starting with the upright session (light pink); M_UPR, males starting with the upright session (light blue); F_INV, females starting with the inverted session (dark pink); M_INV, males starting with the inverted session (dark blue).
